# Acute Appendicitis Presenting as Unusual Left Upper Quadrant Pain

**DOI:** 10.5812/iranjradiol.6326

**Published:** 2013-08-30

**Authors:** Tsung-Ju Chuang, Chun-Wen Chen, Hsin-Yuan Lin, Wen-Hsiu Hsu, Shou-Cheng Wang, Chuan-Chou Tu

**Affiliations:** 1 Department of Internal Medicine, Armed Forces Taichung General Hospital, Taichung, Taiwan; 2 Department of Radiology, Armed Forces Taichung General Hospital, Taichung, Taiwan; 3 Department of Surgery, Armed Forces Taichung General Hospital, Taichung, Taiwan; 4 Department of Gastroenterology & Hepatology, Armed Forces Taichung General Hospital, Taichung, Taiwan

**Keywords:** Appendicitis, Intestinal Malrotation, Familial, Abdomen, Acute, Abdominal Pain

## Abstract

Appendicitis is the most common abdominal disease that requires surgery in the emergency ward. It usually presents as right lower quadrant pain, but may rarely present as left upper quadrant (LUQ) pain due to congenital anatomical abnormalities of the intestine. We report a patient who complained of persistent LUQ abdominal pain and was finally diagnosed by computed tomography (CT) as congenital intestinal malrotation complicated with acute appendicitis. It is important to include acute appendicitis in the differential diagnosis of patients who complain of LUQ abdominal pain. Abdominal CT can provide significant information that is useful in preoperative diagnosis and determination of proper treatment.

## 1. Introduction

The incidence of acute appendicitis in a well-defined population was 1.33/1000 in males and 0.99/1000 in females. The highest incidences in both genders occurred in the second decade of life (2.34/1000) (1). Because the early signs and symptoms of appendicitis are often subtle, patients and clinicians may downplay their importance. Although considered a classic symptom, migratory pain beginning in the periumbilical region and then moving to the right lower quadrant occurs only in 50%-60% of patients with appendicitis (2). Approximately, one-third of the patients with acute appendicitis have pain localized outside the right lower quadrant because of the various positions of the appendix; left-sided pain is rarely observed (3).

Differential diagnoses of left upper quadrant (LUQ) abdominal pain includes angina pectoris, myocardial infarction, pericarditis, esophagitis, gastritis, peptic ulcer, pancreatitis, nephrolithiasis, pyelonephritis, aortic dissection and mesenteric ischemia among other causes (4). Moreover, appendicitis is a rare cause that should not be ignored. We report a patient who complained of persistent LUQ abdominal pain and was diagnosed using abdominal computed tomography (CT) as having congenital intestinal malrotation complicated with acute appendicitis.

## 2. Case Presentation

A 50-year-old man with no significant past medical history was brought to the emergency department because of severe abdominal pain without fever or diarrhea. The pain began diffusely in the morning and later became localized in the LUQ after arrival at the emergency department. On physical examination, his blood pressure was 130/75 mmHg and pulse rate was regular at 78 bpm. Abdominal examination showed localized tenderness over the LUQ without rebound tenderness. Laboratory blood tests revealed leukocytosis (white blood cell count=16, 200×103/µL) with 86% neutrophils. Other tests, including C-reactive protein (CRP), blood sugar, kidney function, liver function and electrolytes were normal. Because of this unexplained and persistent clinical presentation, abdominal CT was performed 3 hours later.

**Figure 1.**
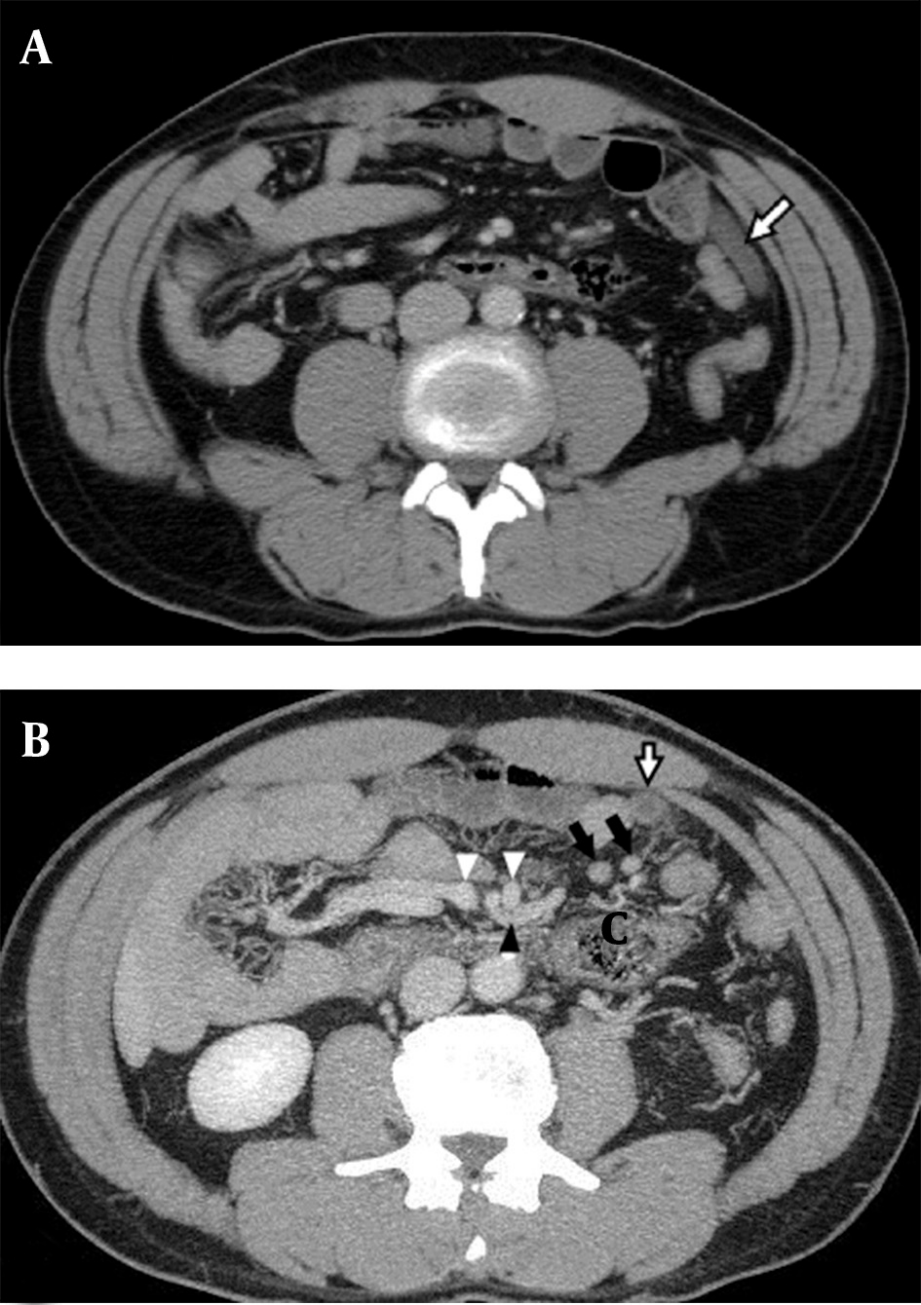
A, The CT image shows a misplaced ascending colon with the cecal base located in the LUQ of the abdomen. Swelling of the appendix (white arrow) (0.8-cm calibre) is seen. B, The maximum intensity projection of axial plane with 20 mm thickness shows swelling of the appendix (white arrow) in the anterior aspect of the cecum (C), with few inflammatory and enlarged lymph nodes (black arrows). The ileocolic artery (black arrow head) arises from the superior mesenteric artery, between the inferior mesenteric vein (right white arrow head) and the superior mesenteric vein (left white arrow head). The ileocolic artery is passing under the inferior mesenteric vein and supplies the cecum in the LUQ of the abdomen.

CT image (Figure 1 A) showed a misplaced ascending colon with the cecal base located in the LUQ of the abdomen and swelling of the appendix (0.8-cm calibre) was seen. The maximum intensity projection of axial plane (Figure 1 B) with 20mm thickness showed swelling of the appendix in the anterior aspect of the cecum with few inflammatory and enlarged lymph nodes. Based on these findings, acute appendicitis was diagnosed and then emergency laparoscopic appendectomy was performed; pathological analysis showed acute suppurative appendicitis and periappendicitis. 

**Figure 2.**
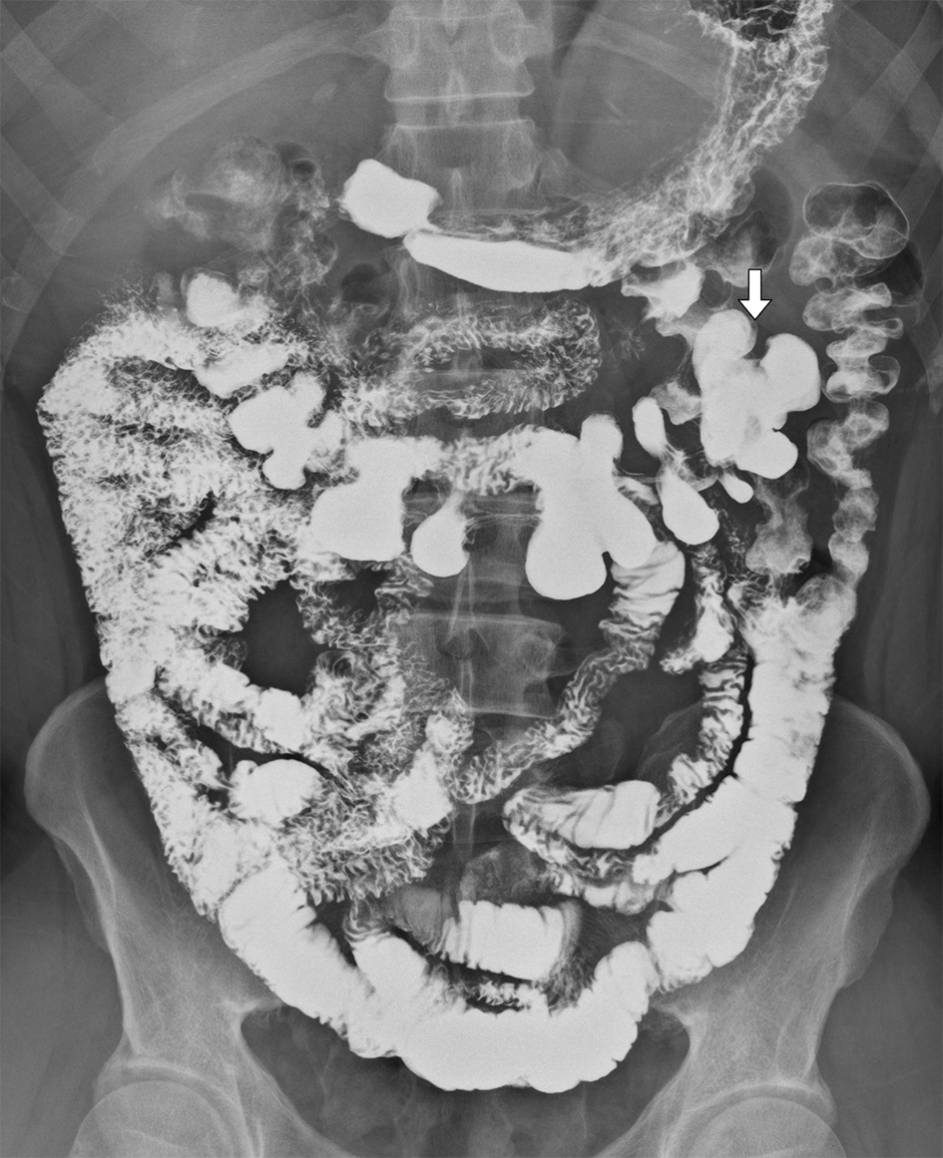
Small bowel GI series using X-ray contrast media. The cecum (arrow) and proximal ascending colon are located in the LUQ of the abdomen, and most of the small intestine is distributed in the right lateral abdomen.

Two weeks postoperatively, a small-bowel GI series using X-ray contrast media was performed because of the abnormal position of the cecum. The cecum and proximal ascending colon were located in the LUQ of the abdomen and most of the small intestine was distributed in the right lateral abdomen (Figure 2). Intestinal malrotation was diagnosed. 

## 3. Discussion

Acute abdominal pain can be a benign self-limiting disease but it can also lead to surgical emergencies. Abdominal pain evaluation requires an approach relying on the likelihood of the disease, patient history, physical examination, laboratory tests and imaging studies (4). Appendicitis is the most common reason for emergency abdominal surgery accounting for 17% of all acute cases (5). However, despite its prevalence, it can be difficult to diagnose, and delayed diagnosis is the main cause of perioperative mortality and morbidity. In its typical presentation, acute appendicitis begins with an ambiguous abdominal discomfort around the epigastric or periumbilical region accompanied by nausea. Several hours later, the pain might shift to the right lower quadrant of the abdomen near McBurney’s point (6). Additionally, fever, psoas sign, rebound tenderness, rigidity and anorexia may be observed (6). The initial laboratory workup always includes a complete blood count with a differential count. Up to 90% of appendicitis cases demonstrate mild-to-moderate leukocytosis. Other studies have shown that neutrophilia (95%) and elevated CRP (97%–100%) are more sensitive indicators. However, these criteria alone are no substitute for a thorough clinical history and examination (6). In our case, the patient complained of LUQ abdominal pain rather than the typical right lower abdominal pain; he had no fever and a normal CRP level. So, appendicitis was difficult to diagnose initially.

LUQ pain is caused by a variety of clinical conditions; therefore, imaging recommendations are not clear-cut. If the patient’s history and physical examination suggest esophageal or gastric problems, endoscopy or an upper gastrointestinal series using x-ray contrast medium is recommended. In other patients with LUQ pain, CT is useful because it provides imaging of the pancreas, spleen, kidneys, intestines and abdominal vasculature. Generally, CT is highly effective in identifying patients with nonspecific abdominal pain who need urgent intervention (7). CT scanning can provide an accurate diagnosis of appendicitis, detect anatomical abnormalities and is frequently considered when local peritoneal signs develop or the clinical symptoms and signs are unresolved during observation. It has been advocated as an appropriate first-line examination for appendicitis with a sensitivity of 100% and specificity of 97% (7). In our case, LUQ tenderness persisted during observation. Although the patient had no fever, blood leukocytosis was found on testing. Intra-abdominal infection could not be excluded and abdominal CT was performed 3 hours after results were obtained from the initial examinations. Fortunately, a nonperforated swollen appendix was accidentally discovered before his condition became severe. Moreover, abdominal CT (Figure 1 B) showed malpositioning of the ascending colon with the cecum (C) base located in the LUQ abdomen and the ileocolic artery arising from the superior mesenteric artery between the inferior mesenteric vein and the superior mesenteric vein. The ileocolic artery is passing under the inferior mesenteric vein and supplies the cecum in LUQ abdomen. Therefore, congenital anatomical abnormalities were strongly suspected. We arranged a small-bowel GI series using X-ray contrast media for the patient to obtain the complete anatomy of the intestines that showed an incompletely rotated duodenal loop with the duodenojejunal junction overlapping the spine (Figure 2) matching the definition of intestinal malrotation (8).

Congenital anatomical abnormalities resulting in left-sided appendicitis are usually caused by situs inversus and midgut malrotation (9). In a study published in 2010, Akbulut et al. reviewed a total of 95 cases of left-sided appendicitis published from 1893 to July 2010. In these cases, 66 patients had situs inversus, 23 had intestinal malrotation (24%) and only 7 had LUQ abdominal pain (7.3%) (9). Intestinal malrotation is a developmental abnormality occurring as a result of nonrotation or incomplete rotation of the primitive loop around the axis of the superior mesenteric artery during weeks 5-10 of fetal life (8). The estimated incidence of intestinal malrotation varies from 0.03%-0.5% for live births. As many as 40% of patients with intestinal malrotation present within the first week of life, 50% of the patients are diagnosed within the first month of life and up to 75% are diagnosed within the first year of life. The remaining 25% of patients present after 1-year of age and into adulthood; many are recognized intraoperatively during other procedures or at autopsy (10). In adults, malrotation may present acutely to the general surgeon with conditions related to the abnormality, such as cecal volvulus and band obstruction (Ladd band), or incidentally, as a result of unrelated abdominal pathology such as acute appendicitis (10).

Briefly, acute appendicitis may rarely present as LUQ pain due to congenital anatomical abnormalities of the colon thus posing a significant diagnostic challenge. Adequate observation and high suspicion are necessary for patients with uncertain clinical features. However, in patients with unresolved clinical symptoms, abdominal CT can provide significant information useful in the preoperative diagnosis and determination of proper treatment.
